# Optimization of glutathione production in batch and fed-batch cultures by the wild-type and recombinant strains of the methylotrophic yeast *Hansenula polymorpha *DL-1

**DOI:** 10.1186/1472-6750-11-8

**Published:** 2011-01-22

**Authors:** Vira M Ubiyvovk, Vladimir M Ananin, Alexander Y Malyshev, Hyun Ah Kang, Andriy A Sibirny

**Affiliations:** 1 Institute of Cell Biology NAS of Ukraine, Drahomanov Street, 14/16, Lviv, 79005 Ukraine; 2 Korea Research Institute of Bioscience and Biotechnology, Daejeon, 305-333, Korea; 3 Department of Life Science, Chung-Ang University, Heukseok-dong, Dongjak-gu, Seoul, 156-756, Korea; 4 University of Rzeszow, Cwiklinskiej 2, Rzeszow 35-601 Poland

## Abstract

**Background:**

Tripeptide glutathione (gamma-glutamyl-L-cysteinyl-glycine) is the most abundant non-protein thiol that protects cells from metabolic and oxidative stresses and is widely used as medicine, food additives and in cosmetic industry. The methylotrophic yeast *Hansenula polymorpha *is regarded as a rich source of glutathione due to the role of this thiol in detoxifications of key intermediates of methanol metabolism. Cellular and extracellular glutathione production of *H. polymorpha *DL-1 in the wild type and recombinant strains which overexpress genes of glutathione biosynthesis (*GSH2*) and its precursor cysteine (*MET4*) was studied.

**Results:**

Glutathione producing capacity of *H. polymorpha *DL-1 depending on parameters of cultivation (dissolved oxygen tension, pH, stirrer speed), carbon substrate (glucose, methanol) and type of overexpressed genes of glutathione and its precursor biosynthesis during batch and fed-batch fermentations were studied. Under optimized conditions of glucose fed-batch cultivation, the glutathione productivity of the engineered strains was increased from ~900 up to ~ 2300 mg of Total Intracellular Glutathione (TIG) or GSH+GSSG_in_, per liter of culture medium. Meantime, methanol fed-batch cultivation of one of the recombinant strains allowed achieving the extracellular glutathione productivity up to 250 mg of Total Extracellular Glutathione (TEG) or GSH+GSSG_ex_, per liter of the culture medium.

**Conclusions:**

*H. polymorpha *is an competitive glutathione producer as compared to other known yeast and bacteria strains (*Saccharomyces cerevisiae, Candida utilis, Escherichia coli, Lactococcus lactis *etc.) with good perspectives for further improvement especially for production of extracellular form of glutathione.

## Background

Tripeptide glutathione (γ-glutamyl-L-cysteinyl-glycine) is the most abundant non-protein thiol compound of the most living organisms that protects cells from nutritional, environmental and oxidative stresses [1]. More than 90% of microbial, plant and mammalian cell glutathione is present in the reduced form, designated as GSH [2]. Both thiol GSH and disulfide form of oxidized glutathione, GSSG, are widely used in medicine and cosmetic industry, as well as food additives [3,4]. As an active ingredient of food, drugs and cosmetic products, GSH could alleviate harmful oxidative processes, scavenge toxic compounds at different kinds of human intoxications and strengthen whitening, skin repair anti-aging effect. Oxidized form of glutathione, GSSG, could be used as cryoprotector, immunomodulator etc. [5,6]. Thus, production of glutathione has great commercial importance.

In spite of the expanding commercial demand for glutathione, its application is restricted due to the high production costs. GSH and GSSG could be produced by enzymatic methods (expansive and unprofitable) and by fermentation using natural or engineering microorganisms (yeasts *Saccharomyces cerevisiae *and *Candida utilis*, bacteria *Escherichia coli *and *Lactococcus lactis *etc.) [3,4]. As a rule, microbial GSH overproduction is limited by mechanisms of feedback inhibition by GSH of the activity of the first and rate-limiting enzyme of GSH biosynthesis, gamma-glutamylcysteine synthetase (GCS) as well as GSH-exerted repression of the structural genes coding for GCS [2-4]. Thus, the search and/or construction of new and efficient glutathione producers as well as optimization of conditions of glutathione biosynthesis would assist in its bringing to market for the improvement of quality of food, cosmetic and pharmaceutical products.

During the last decades the methylotrophic yeast *Hansenula polymorpha *from three genetic lines, DL-1, CBS4732, NCYC495, have gained increasing interest, both for basic research and biotechnological applications, which include studying the mechanisms of thermotolerance, peroxisome homeostasis, production of numerous heterologous proteins and high-temperature alcoholic fermentation [7-12]. The industrial relevance of *H. polymorpha *is mostly explained by several technologically interesting features. This yeast grows to very high cell densities in bioreactors, possesses strong regulatory and constitutive promoters and consequently gives high product yields. Like *S. cerevisiae*, *H. polymorpha *is characterized by simple cultivation mode in inexpensive growth media, well established genetic tools and experience on industrial cultivation and scaling-up. *H. polymorpha *is considered as GRAS organism, it does not harbour pyrogens, pathogens or viral inclusions. In addition, completed genome sequencing [13,14], established proteome and transcriptome databases [15,16] makes *H. polymorpha *suitable organism for metabolic engineering in order to modify and improve particular biosynthetic pathways [10-12].

*H. polymorpha *is regarded as a rich source of glutathione, due to the role of this tripeptide in detoxifications of key intermediates of methanol metabolism, formaldehyde, as well as hydrogen peroxide and alkyl hydroperoxides, which are accumulated during methylotrophic growth [17-19].

In our previous study, we estimated physiological role of some *H. polymorpha *genes involved in GSH homeostasis: *H. polymorpha GSH2 *gene, *HpGSH2*, a homologue of *S. cerevisiae GSH1 *gene coding for GCS, [20-22]; *H. polymorpha GGT1 *gene, homologue of *S. cerevisiae CIS2/ECM38 *gene encoding for gamma-glutamyltranspeptidase [23]; *H. polymorpha MET4 *gene, *HpMET4*, similarly *to S. cerevisiae MET4 *gene involved in global sulfur regulation [24,25]. We identified GSH as the sole cadmium ion chelator in *H. polymorpha *[26]. Meantime, until now, biotechnological potential of *H. polymorpha *in production of glutathione was not elucidated. In the present study, we studied glutathione producing capacity of *H. polymorpha *DL-1 wild-type strain and several recombinant strains with overexpression of the first gene in glutathione synthesis *HpGSH2*, depending on parameters of cultivation (dissolved oxygen tension, pH, stirrer speed), carbon substrate (glucose, methanol) and type of overexpressed genes of GSH biosynthesis in recombinant strains during batch and fed-batch fermentations. Under optimized conditions of glucose fed-batch cultivation, engineered strains accumulated more than 2250 mg of Total Intracellular Glutathione (TIG) or GSH+GSSG_in_, per liter of culture medium, which slightly exceeds the known before maximal glutathione production in *S. cerevisiae *of 2020 mg/L (however, last number was obtained with addition of amino acids to cultural medium, which increases the costs of the aimed product) [27]. One of constructed by us *H. polymorpha *recombinant strains was promising producer of the extracellular glutathione in methanol medium as accumulated 5-times higher titer of Total Extracellular Glutathione (TEG) or GSH+GSSG_ex_, as compared to best level achieved before for yeasts [3].

## Results and Discussion

### Growth and glutathione production of *H. polymorpha *DL-1 wild-type strain cultivated in fermenter batch cultures with glucose at different modes of pH control and aeration

To choose the most glutathione productive strain for further work, we compared intra- and extracellular GSH+GSSG levels (hereinafter - GSH+GSSG_in _or TIG and GSH+GSSG_ex _or TEG, respectively) in *H. polymorpha *wild-type strains DL-1L, CBS4732L and NCYC495L from three genetic lines (Tables 1, 2) [28] using simple enzyme assay that does not differentiate reduced and oxidized forms of glutathione, as both have biotechnological application.

**Table 1:** Strains of *H. polymorpha *used in the present study

Designation	Genotype or relevant features	Reference or source
**Wild type strains**		
NCYC495L	*leu1-1*	Laboratory collection
CBS4732L (A16)	*leu2*	Laboratory collection
DL-1L	*leu2*	Laboratory collection
DL-1	*leu2::LEU2 *(plasmid vector pGLG61)	This study
**Recombinant strains of DL-1**		
*mcGSH2*	*leu2:: LEU2::mcGSH2 *(pGLG61-*HpGSH2*)	This study
*MOXp-GSH2*	*leu2::LEU2::MOXp-GSH2 *(pGLG61- *HpMOXp::HpGSH2*)	This study
*mcMET4*	*leu2:: LEU2::mcMET4 *(pGLG61-*HpMET4*)	Laboratory collection

**Table 2:** Maximal biomass and glutathione (TIG and TEG) production of *H. polymorpha *strains at different conditions of fermentation

Strains	Conditions of fermentation			TIG				TEG	Biomass
				
				nmol/mg protein	% w/w	mg/g of dry cells	mg/L	mg/L	Dry cells g/L
Wild-types NCYC495L				177 ± 1.5	0.6	6	20 ± 0.15	1 ± 0.05	3.2 ± 0.26
CBS 4732L	Shake-	Glucose	200 rpm (w/o pH control)	380 ± 3.2	0.9	9	24 ± 0.19	1 ± 0.06	2.8 ± 0.19
DL-1L	flask			481 ± 3.9	1.0	10	27 ± 0.21	2 ± 0.09	2.8 ± 0.18

			200 rpm (pH control 5.2)	161 ± 1.49	0.5	5	20 ± 1.4	2 ± 0.19	4.0 ± 0.36
	Batch		600 rpm (- " - " - " - " - )	50 ± 0.45	0.3	3	17 ± 1.19	12 ± 1.10	6.9 ± 0.42
			
Wild type DL-1	Fermenter	Glucose	200 rpm (w/o pH control)	373 ± 3.12	1.1	11	69 ± 6.49	2 ± 0.20	6.1 ± 0.39
			800 rpm (- " - " - " - " - )	269 ± 2.11	0.6	6	49 ± 4.45	17 ± 1.59	7.7 ± 0.45

Wild type DL-1		Methanol	DO-stat 60%	136	0.8	8	676	37	90
Wild type DL-1		Glucose	DO- stat 60%	183	1.2	12	518	22	44
Wild type DL-1		Glucose	DO- stat 30%	315	1.3	13	910	4	72
*mcMET4*	Fed-batch	Glucose	(- " - " - " - " )	318	1.6	16	1318	6	82
*mcGSH2*	fermenter	Glucose	(- " - " - " - " )	755	2.0	20	1532	1	77
*MOXp-GSH2*		Glucose	(- " - " - " - " )	409	3.1	31	2257	13	72
*MOXp-GSH2*		Methanol	DO-stat 60%	243	2.1	21	1053	250	55

So, the strains of *H. polymorpha *derived from the genetic line DL-1 with the highest levels of GSH+GSSG_in _(TIG) as well as GSH+GSSG_ex _(TEG) were used for the next studies (Table 2).

**Figure 1:**
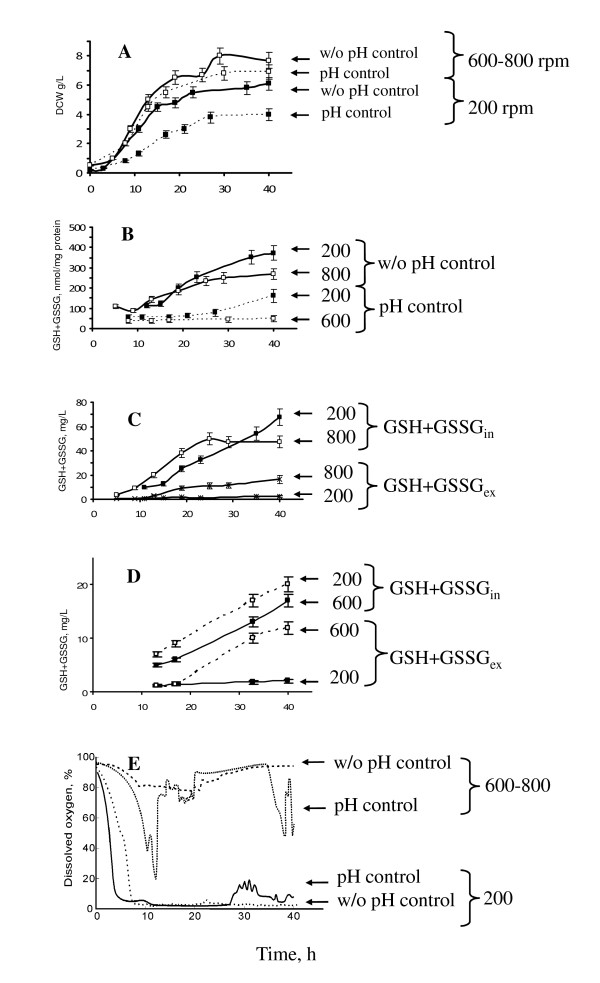
**Growth and glutathione accumulation of *H. polymorpha *DL-1 at fermenter batch cultivations**. **A **- Dry cell weight (DCW); **B**, **C**, **D **- TIG (GSH+GSSG_in_) and TEG (GSH+GSSG_ex_) concentrations, expressed in nmol/mg protein (**B**) and in mg/L culture medium (**C, D**). *H. polymorpha *DL-1 wild type strain was cultivated with glucose at different modes of pH control and aeration: fermentation with pH control at 5.2 (pH control), fermentation without pH control (w/o pH control), aeration at stirrer speed of 200 rpm (200) and at 600 rpm (600); data for TIG and TEG accumulation at different aeration without pH control and with pH control (**C **and **D**, respectively); **E **- data of dissolved oxygen tension in culture media at different mode of aeration and pH control. Values shown are the means of three independent determinations.

The growth and total intracellular glutathione (TIG) content in *H. polymorpha *DL-1 wild-type strain during batch cultivation in fermenter was studied depending on growth parameters, pH and aeration (Figure 1). It was shown that this yeast produced more TIG during cultivation in glucose minimal medium under conditions of spontaneous pH acidification from pH 5.0 to 2.5 as compared to that at mode during pH 5.2 maintenance using an active pH control (Figure 1 - B, C, D; Table 2). It was demonstrated also that TIG was higher at low stirrer speed (200 rpm) than under intensive aeration (600-800 rpm), independently of pH control regime (Figure 1 - B, C, D; Table 2). After 8 h of cultivation at 200 rpm, DO parameter dropped from 94 - 98% to 3 - 4% and was maintained at the latter level till the end of process, in contrast to cultivation at 600-800 rpm, when the excess of oxygen was provided to culture during all process (Figure 1E). Maximal cell biomass concentration was higher under conditions of high saturation with oxygen (shake agitation at 600-800 rpm) as compared to low aeration (200 rpm), independently on the mode of pH control (Figure 1A; Table 2).

It should be noted that the level of Total Extracellular Glutathione (TEG) GSH+GSSG_ex _relative to that of TIG (GSH+GSSG_in_) became significant in the cases of high agitation (600-800 rpm), independently on pH control mode (Figure 1 - C, D). To explain these results, one may suggest that supply cells with more oxygen at 600-800 rpm provided more optimal growth conditions as we observed increase in biomass concentration. Oxygen-limited yeast cells cultivated at low stirrer speed, 200 rpm, most probably are suffered from metabolic stress induced by oxygen limitation. This stress could reduce growth activity and turned on cellular protection mechanisms including glutathione biosynthesis [29]. The reasons of GSH+GSSG_ex _changes depending on oxygen level remain obscure.

### Biomass and glutathione production by *H. polymorpha *DL-1 wild type strain under fed-batch fermentation in glucose minimal medium under different modes of aeration

**Figure 2:**
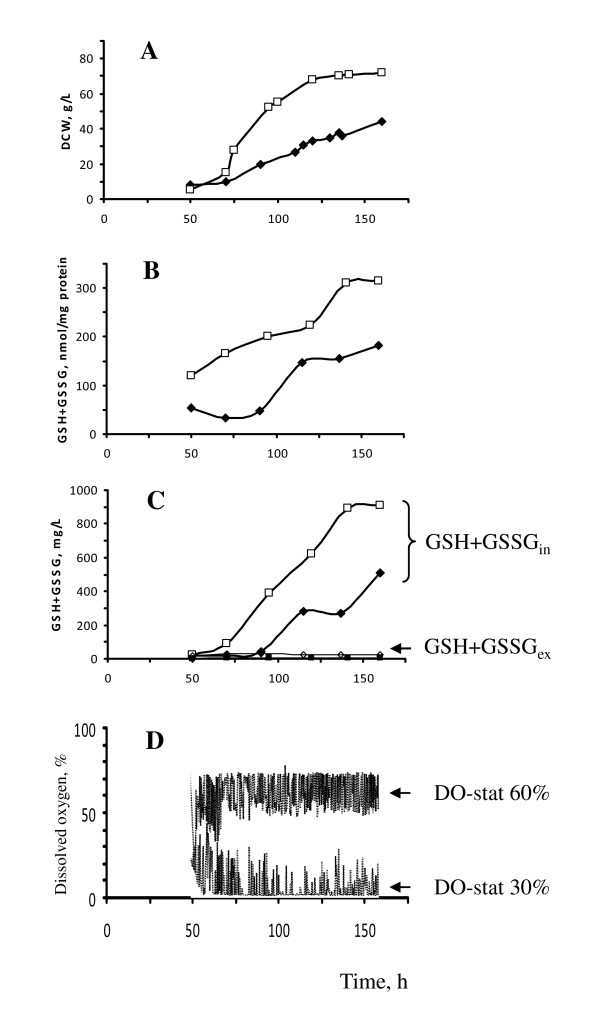
**Growth and glutathione accumulation of *H. polymorpha *DL-1 at fermenter fed-batch cultivation**. **A **- Dry cell weight (DCW); **B**, **C **- TIG (GSH+GSSG_in_) and TEG (GSH+GSSG_ex_) concentrations, expressed in nmol/mg protein (**B**) and in mg/L culture medium (**C**) of *H. polymorpha *DL-1 wild type strain cultivated under different dissolved oxygen tension (DO) set point control of glucose feeding: DO-stat 30% (open square) and DO-stat 60% (filled rhombus). **D **- data of dissolved oxygen tension in culture media at different mode of aeration. Values shown are the means of two independent determinations.

We compared biomass and glutathione producing capacities of *H. polymorpha *DL-1 wild-type strain under different dissolved oxygen (DO) saturation conditions, DO-stat 30% and DO-stat 60%, provided in fed-batch cultures in glucose minimal medium (see Materials and Methods) (Figure 1D). Similarly to batch culture cultivation, we observed elevated level (~2-3 times) of TIG content during fermentation at low oxygen supply, DO-stat 30%, as compared to those at DO-stat 60% (Figure 2 - B, C). Unexpectedly, enhanced oxygen supply (DO-stat 60%) caused reduction of biomass yield of fed-batch cultivated wild-type cells in glucose-containing minimal medium (Figure 2A). We hypothesize that yeast cells supplied with enhanced oxygen suffered from excess of reactive oxygen species. It is known that the lasts could suppress growth activity and trapped accessible for measurement glutathione in the reactions of S-glutathiolation of protein and conjugation with product of lipid peroxidation [30,31].

In the meantime, intensification of oxygen supply led to slight increase in TEG level (Table 2). The maximal concentration of TEG by the wild-type cells was negligible and comprised 4-22 mg/L as compared to maximal TIG content ~900 mg/L achieved under conditions of low oxygen supply, DO-stat 30% under fed-batch conditions for the same cells (Figure 2C; Table 2).

### Biomass and glutathione production by *H. polymorpha *DL-1 recombinant strains at fed-batch fermentation in glucose minimal medium

**Figure 3:**
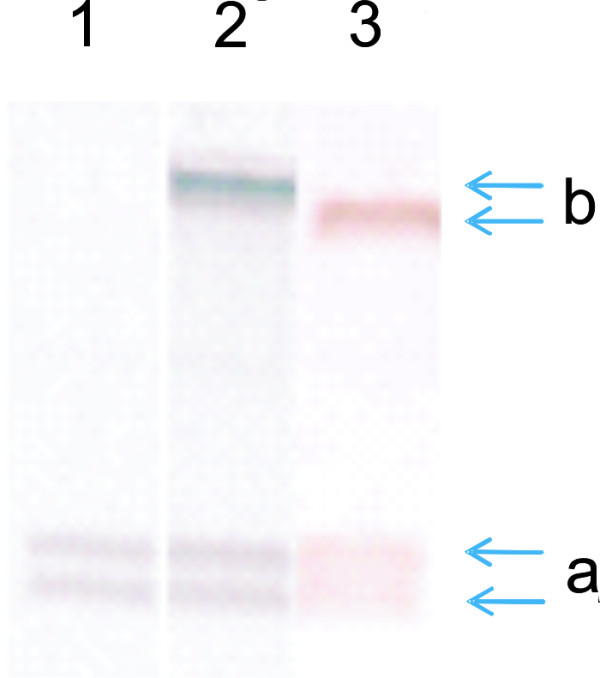
**Southern blot analysis of copy number of *HpGSH2 *and *HpMET4 *genes in *mcGSH2 *and *mcMET4 *transformants**. Copy number of integrated into chromosomal DNA of *H. polymorpha *DL-1L wild type strain (1), *mcGSH2 *(2) and *mcMET4 *(3) transformants pGLG61-*HpGSH2 *and pGLG61-*HpMET4 *plasmids bearing *LEU2 *gene. The DNA probes to *LEU2 *gene discovered mutated *LEU2 *gene (a) in all tested strains and introduced *LEU2 *gene based on indicated plasmids only in transformants (b).

We hypothesized that accumulation of elevated TIG level can be achieved in recombinant *H. polymorpha *strains which overexpress this tripeptide. Consequently, we have studied TIG production in several *H. polymorpha *recombinant strains which harbored the additional copies of *HpGSH2 *gene. As it was revealed earlier, *H. polymorpha GSH2 *gene, *HpGSH2*, a homologue of *S. cerevisiae GSH1 *gene, encoding GCS, the first enzyme of GSH biosynthesis, is essential for yeast growth and stress reply [20,21]. One of the obtained in the present study recombinant strains designated as *mcGSH2 *transformant possessed 2-3 copies of *HpGSH2 *expression cassette under native *H. polymorpha GSH2 *promoter (Figure 3). Another recombinant strain, named as *MOXp-GSH2 *transformant, acquired *HpGSH2 *expression cassette under control of the strong promoter of *H. polymorpha *alcohol oxidase *MOX *gene. The *MOX *promoter is known to be regulated in *H. polymorpha *by mechanisms of glucose repression-derepression and methanol induction [32]. We also studied transformant named as *mcMET4 *that obtained 2-3 additional copies of *H. polymorpha MET4 *gene (Figure 3), putative homologue of *S. cerevisiae MET4 *gene involved in biosynthesis of GSH and its precursor, cysteine [24,25].

**Figure 4:**
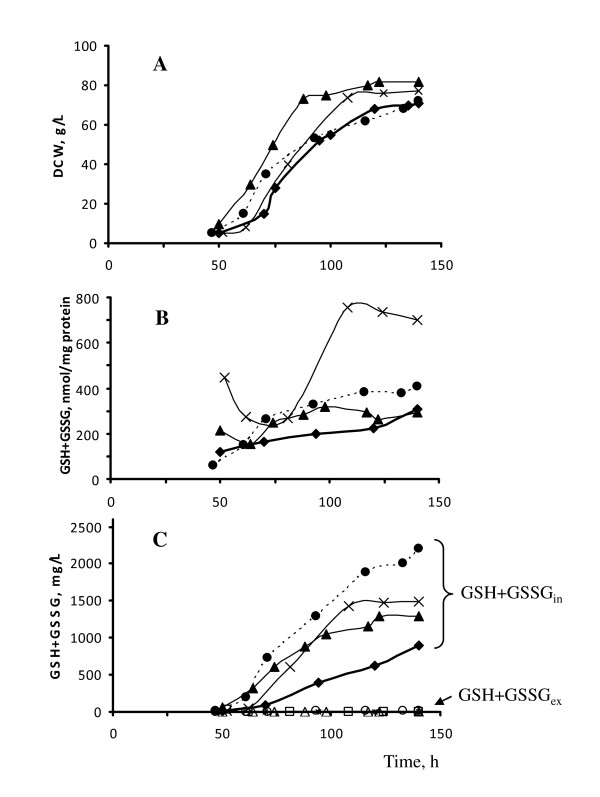
**Growth and glutathione accumulation of *H. polymorpha *DL-1 recombinant strains at glucose-feeding fermenter cultivation**. **A **- Dry cell weight (DCW); **B**, **C **- TIG (GSH+GSSG_in_) and TEG (GSH+GSSG_ex_) concentrations, expressed in nmol/mg protein (**B**) and in mg/L culture medium (**C**) of *H. polymorpha *DL-1 wild type (rhombus) and recombinant strains: *MOXp-GSH2 *(cycle), *mcGSH2 *(cross), *mcMET4 *(triangle) at fed-batch cultivation in glucose minimal medium under condition of DO-stat 30%. TEG concentration is indicated with open symbols **(C)**. Values shown are the means of two independent determinations.

All indicated strains displayed the growth kinetics under fed-batch fermentation process at DO-stat 30% in glucose minimal medium very similar to that of the wild-type strain, except slightly elevated maximal biomass concentration of *mcGSH2 *and *mcMET4 *recombinant strains (by 8% and 13%, respectively) (Figure 4A). If to express glutathione/protein ratio, the highest TIG concentration was found in *mcGSH2 *transformants (Figure 4B). However, when we compared TIG concentrations as the mass percentage of total glutathione to the total dry cell weight (%) or per Liter of culture medium (mg/L), the best TIG producer appeared to be the *MOXp-GSH2 *recombinant strain (Figure 4C; Table 2). We explained such data discrepancy by differences in protein content of the compared strains. The strains in decreasing order of TIG concentrations (%) can be ranged in the following order: *MOXp-GSH2 *- 3.1%, *mcGSH2 *- 2.0%; *mcMET4*- 1.57%; wild type -1.25%. The same order of strains was if to express TIG concentration in mg/L: *MOXp-GSH2 *- 2257 mg/L; *mcGSH2 *- 1532 mg/L; *mcMET4*- 1318 mg/L; wild type -910 mg/L.

Thus, introduction of additional copies of putative *HpMET4 *gene into the genome of recipient strain 1.5-fold increased the TIG level in *mcMET4 *transformant, probably, due to activation of biosynthesis of GSH precursor, cysteine.

*mcGSH2 *transformant displayed only 1.7 times enhanced TIG level as compared to that in the wild-type strain in spite of obtaining of 3-4 copies of *HpGSH2 *gene. We have found the *HpGSH2 *gene overexpression in *mcGSH2 *transformant cultivated in medium without any carbon substrate (Figure 4). Such conditions resembled fed-batch cultivation where glucose concentrations was maintained at the level lower than 0.01-0.05%. It seems that GSH excess restricted glutathione overproduction in our recombinant strains by mechanism of feedback inhibition of GCS activity.

**Figure 5:**
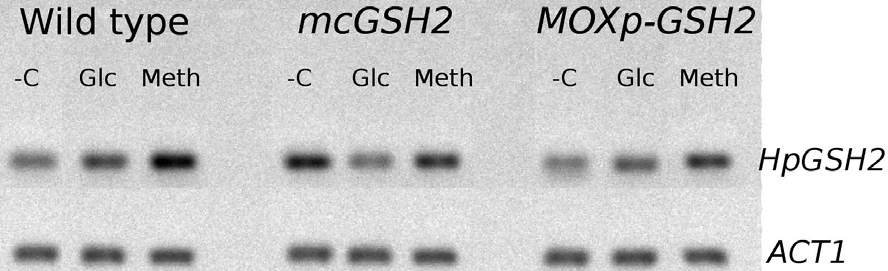
**Transcript level of *HpGSH2***. RT-PCR was performed on RNA samples using *HpGSH2 *primers from cultures of wild type, *mcGSH2 *and *MOXp-GSH2 *recombinant strains shifted for 6 hours from glucose medium to glucose (Glc), methanol (Meth) or with no carbon added (-C) media. As loading control transcript levels of actin (*ACT1*) were analysed.

Nevertheless we did not confirm regulated from *MOX *promoter expression of *HpGSH2 *gene (data of RT-PCR) in the recombinant strain that obtained such construct. Mode of expression of *HpGSH2 *gene from *MOX *promoter was the same as in the wild-type strain (Figure 5). So, 2.5 times improvement of glutathione production in *MOXp-GSH2 *transformant could be explained by insertion of cassette into some regulatory locus important for GSH biosynthesis or under unknown promoter.

Concentration of the TEG (GSH+GSSH_ex_) by the studied wild type and recombinant strains was negligible: 2-13 mg/L as compared to their TIG level comprising of 910-2257 mg/L (Figure 4C; Table 2).

### Biomass and glutathione production by *H. polymorpha *DL-1 wild type and *MOXp-GSH2 *recombinant strains during fed-batch fermentation in methanol minimal medium

*HpGSH2 *transcript analysis revealed methanol-dependent induction of this gene expression in all studied strains compared to that of glucose incubated yeast cells (Figure 5). Nevertheless, we did not observe strong increase of TIG concentration in methanol-feeding wild type cells (DO-stat 60%) relative to that of glucose-feeding cells (DO-stat 60%) (Table 2). Methanol fed-batch fermentations study was carried out only under DO-stat 60% that provides the highest biomass yield of *H. polymorpha *compared to DO-stat 30% [33,34]. Moderate TIG concentrations (expressed in nmol/mg protein or %) during methanol feeding process was partially compensated by high maximal concentration of biomass (90 g/l). We suggested that application of oxygen control during methanol feeding under fed-batch cultivation prevents the over-feeding of methanol and overaccumulation of toxic intermediates that could additionaly induce GSH biosynthesis [18].

**Figure 6:**
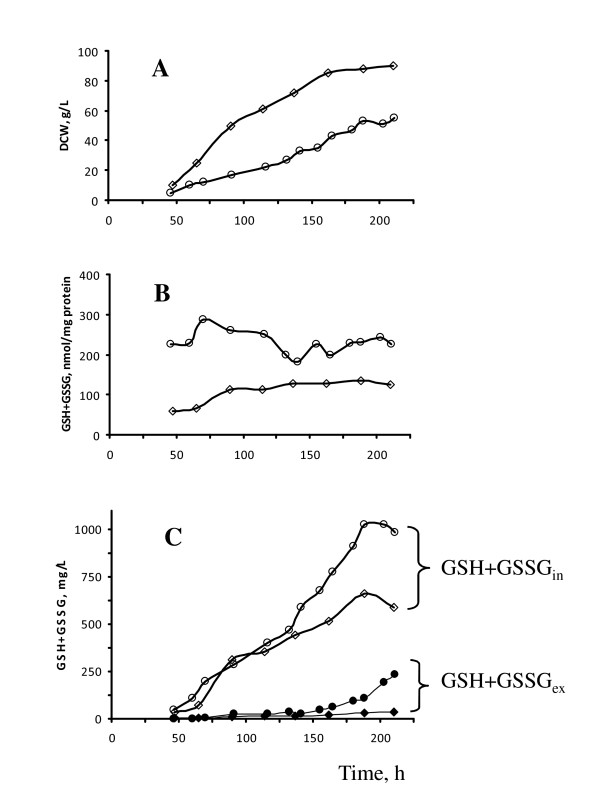
**Growth and glutathione accumulation of *H. polymorpha *DL-1 recombinant strain at methanol-feeding fermenter cultivation**. **A **- Dry cell weight (DCW); **B**, **C **- TIG (GSH+GSSG_in_) and TEG (GSH+GSSG_ex_) concentrations, expressed in nmol/mg protein (**B**) and in mg/L culture medium (**C**) of *H. polymorpha *DL-1 wild type (rhombus) and recombinant strain *MOXp-GSH2 *(cycle) at fed-batch cultivation in methanol minimal medium under condition of DO-stat 60%. TEG concentration is indicated with filled symbols **(C)**. Values shown are the means of two independent determinations.

Studied here *MOXp-GSH2 *recombinant strain was characterized by normal growth in glucose fed-batch cultures with minimal medium (Figure 4A) but displayed retarded growth in methanol as the sole carbon source as compared to the wild-type strain (Figure 6A). During growth in methanol fed-batch culture, *MOXp-GSH2 *recombinant strain accumulated about 2-2.5 fold more TIG (expressed in nmol/mg protein) than the parental strain (Figure 6B; Table 2). The concentration of TIG (expressed in mg/L) in this transformant two-fold exceeded that produced by the wild-type strain only in the last period of cultivation (Figure 6C). The level of TEG in *MOXp-GSH2 *transformant grown in methanol was found to be the highest (250 mg/L) among all tested strains and conditions (Table 2), though it was significantly elevated only at the late fermentation period (Figure 6C).

Reduced ability of *MOXp-GSH2 *recombinant strain to grow in methanol medium could be caused by inhibition of key enzyme of formaldehyde assimilation, dihydroxyacetone synthetase, by excess of GSH [35] or partial impairment of *MOX *gene expression as the result of *MOXp-GSH2 *expression vector integration into the chromosomal *MOX *locus [36].

So, high-cell-density cultivation of *H. polymorpha *DL-1 wild type strain led to accumulation of large amount of glutathione under dual control of substrate feeding (exponential feeding rate mode combined with feedback control of substrate feed by DO set point using on-off regime). Glucose feeding controlled by DO-stat 30% was found to promote the biomass and TIG concentration of the wild-type strain to 72 g/L and 910 mg/L, respectively (against concentration of biomass and TIG of 44 g/L and 518 mg/L, respectively, revealed at DO-stat 60%). Using the methanol feeding control by DO-stat 60%, the biomass and TIG levels of the wild-type strain reached 90 g/L and 676 mg/L, respectively. Genetic engineering of sulfur metabolism and glutathione biosynthetic pathway improved the productivity of glutathione synthesis in *H. polymorpha *recombinant strains 1.3-2.5 times. TIG, TEG and biomass concentrations of the best glutathione-producing recombinant strain, *MOXp-GSH2*, under methanol fed-batch cultivation comprised 1053 mg/L, 250 mg/L and 55 g/L, respectively, and at glucose feeding process these parameters were 2257 mg/L, 13 mg/L and 72 g/L.

One of the best published results on GSH and biomass level for high-GSH-accumulated strain of *S. cerevisiae *G-14 was reported to be 1620 mg/L and 140 g/L, respectively, after 52 h of high-cell-density cultivation with control of glucose feeding by respiratory quotient and ethanol concentration. Optimized process for this strain with addition of amino acids resulted in the glutathione concentration elevation to 2020 mg/L [27]. Genetically modified strains of the methylotrophic yeast *H. polymorpha *DL-1 studied in the present work, appeared to be competitive glutathione producer with good perspectives for further improvement.

To develop the process of glutathione production based on described here *H. polymorpha *producers, we plan to further optimize growth conditions to increase biomass concentration. Other approach we plan to use will be based on engineering GCS protein for impairment of feedback inhibition normally exerted by glutathione. GCS feedback inhibition engineering will be carried out on the isolated in our laboratory *H. polymorpha *mutants defective both in glutathione uptake and secretory pathways which we found to possess elevated glutathione pool (V. Ubiyvovk et al., unpublished data). It is known that *S. cerevisiae *mutants defective in glutathione transport and in secretory pathways overaccumulate glutathione in the medium [37].

## Conclusions

This study has estimated biothechnological potential of the methylotrophic yeast *H. polymorpha *to produce antioxidant glutathione used as medicine, food additives and in cosmetic industry. Our data showed that recombinant strains of *H. polymorpha *with genetically engineered pathway of global sulfur and glutathione metabolism accumulated the highest to our knowledge titre of intracellular and extracellular glutathione under optimized conditions. We consider *H. polymorpha *as the yeast with good perspectives for further improvement especially for production of extracellular form of glutathione.

## Methods

### Microorganisms and cultivation

Strains of the methylotrophic yeast *H. polymorpha *used in this study are listed in Table 1. For shake-flasks and batch cultivation, strains were grown in YNB medium: 0.17% (w/v) yeast nitrogen base without amino acids, 0.5% (w/v) (NH_4_)_2_SO_4_, 2% (w/v) glucose or 1% glycerol. For shake-flasks cultivation it was used shaking diameter of 15 cm. For fed-batch fermentations it was used synthetic salt medium (per litre): (NH_4_)_2_SO_4 _- 89.0 g, KH_2_PO_4 _- 28.5 g, MgSO_4 _⋅ 7H_2_O - 6.0 g, trace elements solution - 5 ml, biotin - 0.5 mg, thiamine hydrochloride - 20 mg. Glucose solution of 500 g/L and 100% methanol were used as the feed media. For transformants stabilization it was used YPD medium: 1% yeast extract, 2% peptone, 2% glucose.

### Construction of recombinant *H. polymorpha *strains with integrated multicopy plasmids harbouring genes of GSH biosynthesis

Standard DNA manipulations were performed as described in Sambrook et al. [38]. Shuttle vector pGLG61, which bears *LEU2 *selectable marker and gives tandem integrated and mitotically stable copies near the end of the chromosome, was used [39].

pGLG61-*HpGSH2 *plasmid bearing *H. polymorpha GSH2 *gene, homologue of *S. cerevisiae GSH1 *gene, was constructed by subcloning of *Sma*I-*Hpa*I fragment of pG2 plasmid [21] (1623 bp upstream and 2694 bp downstream regions of ATG codon of *H. polymorpha *CBS4732 chromosomal *HpGSH2 *gene) into *Not*I digested and blunted pGLG61 vector.

Plasmid with *HpGSH2 *driven by strong promoter of *H. polymorpha *alcohol oxidase *MOX *gene, pGLG61-*HpMOXp::HpGSH2*, was constructed by replacement of human serum albumin (*HSA*) open reading frame (ORF) from pGLG61-*pMOX::HSA *plasmid, derived from pYHSA12(+) [36] to ORF of *HpGSH2 *CBS4732 (1792 bp).

Yeast cells of *H. polymorpha *DL-1 *leu2 *were transformed with appropriate plasmids by electroporation as described earlier [21]. Leu^+ ^transformants that acquired plasmids (pGLG61, pGLG61-*HpGSH2 *or pGLG61-*HpMOXp::HpGSH2*) were isolated and stabilized by multiple (2-3) passage of transformant culture from YPD to YNB media to integrate plasmids into the genome. Copy number of integrated into chromosomal DNA plasmids was estimated with Southern blot analysis. For that total chromosomal DNA was digested with *Cla*I endonuclease, fractionated on 0.8% agarose gel, and then capillary transferred onto a nylon membrane (Schleicher a.Schull GmbH, Dassel, Germany). The DNA probes for Southern blot analysis were labelled with digoxigenin (DIG), using DIG-labelling kit (Roche Diagnostics, Mannheim, Germany).

### Reverse transcription (RT)-PCR analysis

For *HpGSH2 *transcript analysis yeast cells were grown in YNB medium (1% glucose) till the middle of the logarithmic phase. Pelleted cells of each studied strain were once washed with water, resuspended in YNB medium to OD-1.0 (600 nm) without any carbon substrate, split into three equal portions and incubated in the flasks at 37°C on rotary shaker (200 rpm) during 1 hour. Later to one out of three flasks of cells suspension it was added glucose (2%) or methanol (0.5%) or was not added anything for the further incubation during 6 hours.

Total RNA was extracted from yeast cells using the Trizol method (Invitrogen Corporation, Carlsbad, California, USA) following the manufacturer's protocol. RNA was quantified using UV spectrophotometry and diluted in RNAse-free water. Single-stranded cDNA was synthesized using MuLV reverse transcriptase (First Strand cDNA Synthesis Kit, Fermentas, Vilnius, Lithuania). Quantitative RT-PCR analysis was carried out using gene-specific primer pairs and cDNA as a template. The following primer pairs were used: Ax21F 5'-ATCCACAGCGCAATACATACG-3'/Ax22R 5'-TTAACCATTGACCCAGTTCGG-3' for the 3' fragment of *H. polymorpha GSH2*; AxF19 (5'-AGATTCAGAGCCCCAGAAG-3') and AxR20 (5'-GCAATACCTGGGAACATGG-3') for the 3' fragment of *H. polymorpha *ORF of the *ACT1 *gene. Sequence of the gene *ACT1 *was taken from the *H. polymorpha *database [14].

### Fermentation techniques

Inoculum culture of *H. polymorpha *was prepared by 24 h cultivation in shake-flasks in YNB medium supplemented with glucose. All kinds of fermentations were carried out in BioFlo III bench-top fermenter (New Brunswick Scientific Co. Inc., USA) equipped with standard probes (pH, dissolved oxygen tension, DO, and temperature), with 2.5 liters of media (YNB or synthetic salt media) at 37°C without pH control or at pH 5.2 (maintained by addition of 30% NH_4_OH or 2 M HCl) and with aeration 1 vvm and 200 - 1000 rpm of stirrer speed.

The fermentations were computer controlled and monitored. High cell density cultivation experiments were carried out as fed-batch fermentations using dual control algorithms of carbon source feeding. Dual control algorithms were programmed using AFS-BioCommand control diagrams, a pictorial function-block language. In fed-batch fermentations set point of substrate (100% methanol or 50% glucose) feed rate changed exponentially [33]. This control mode was combined with feedback control of substrate feed by dissolved oxygen tension (so called DO-stats) using on-off regime. In other words, current value of substrate feed rate was equal to exponentially increasing set point of substrate feed rate till the moment when current value of controlling parameter (DO_CV_) was passed through the DO set point (DO_SP_). After return DO_CV _to the DO_SP _predetermined exponential feeding control continued operation. Finally fed-batch processes were represented as pulsing feeding with exponentially increased magnitude of pulsation [34,40].

### Fed-batch fermentation on glucose

It has been done two modes of glucose feeding, using DO-set points 30% and 60% (DO-stat 30% and DO-stat 60%), both modes were based on dual control algorithm. DO-stat 30% (mode I) was chosen to create reduced oxygen concentration and DO-stat 60% (mode II) was realized for maintenance of oxygen saturation condition. Both kinds of fermentations (with mode I or II) were started as simple batch processes on 2% glucose at 200 rpm or 600 rpm of stirrer speed, respectively. Glucose feeding was started after stoppage of batch culture growth and followed with gradual increase of stirrer speeds: for fed-batch fermentation of mode I - from 200 to 800 rpm during 24 h and for mode II - from 600 to 800 rpm during 12 h.

Mode I of fed-batch was applied for cultivation of all tested strains. Mode II of process was used for cultivation of wild type only.

### Fed-batch fermentation on methanol

Methanol feeding was started after short batch phase of growth on 1% glycerol (duration 15-17 h) in order to accumulate cell biomass sufficient for DO-response to the methanol addition. Methanol feeding was begun only after complete consuming of glycerol which indicated by sharp increasing of DO level. Next 24 h of cultivation were carried out at gradual increase of stirrer speed from 600 to 1000 rpm to avoid strong oxygen limitation. Dual control of methanol feeding with DO-set point 60% (DO-stat 60%) provided permanent biomass growth. At the time when no changes of biomass concentration observed process was terminated.

### Analytical methods

Cell concentration was evaluated optically at 600 nm using spectrophotometer Ultrospec 2000 (Pharmacia, Sweden), and by dry cell weight (DCW, g/L). Dry cells were prepared by centrifuging 5 ml of the culture medium at 1000 × g for 5 min, followed by drying at 105°C for 4 h. The concentration of glucose in cultivation medium was measured using glucose oxidase method with a glucose assay kit (Sigma-Aldrich Corporation, St. Louis, Missouri, USA).

The total glutathione concentration (GSH+GSSG) was measured in culture medium supernatant and in the cell-free extracts. The lasts were prepared by yeast cells vortexing in Eppendorf microtubes at 4°C for 20 min with 0.1 M potassium-phosphate buffer, KPB, pH 7.5 and glass beads, 425-600 μm, in the ratio 1:1:1 (v/v/v) followed with centrifugation of the mixture at 4°C for 20 min at 20000 × g. Protein was determined using Bio Rad DC protein assay in microplate reader at 690 nm.

Total Intracellular Glutathione (TIG) GSH+GSSG content was quantified by a modification of the standard recycling assay based on the reduction of 5,5-dithiobis-(2-nitrobenzoic acid) (DTNB) in the presence of glutathione reductase and NADPH [41]. Procedure was adapted to 96-well plates: each well contained 140 μl of 0.2 mM NADPH, 20 μl of glutathione reductase solution, 0.4 U/ml (both solutions prepared on 0.05 mM KPB pH 7.5 containing 5 mM EDTA) and 20 μl of GSH standard solution (100, 50, 25, 12.5, 6.25, 3.125 and 1.56 μM ) or 20 μl sample solution (10-100 times-diluted extracts). Reaction was started with 20 μl of 3 mM DTNB (promptly added by multipipetman). Microplate was incubated at room temperature for 10-20 min and the absorbance of each well was read at 405 nm using microplate reader. Usually (GSH+GSSG) concentration was expressed in nmoles/mg protein in cell-free extracts or in mg/L culture medium; cellular (GSH+GSSG) content - in % of dry cell weight or mg/g dry cells.

## Abbreviations

GSH: reduced form of tripeptide glutathione; GSSG: disulfide form of oxidized glutathione; TIG: Total Intracellular Glutathione or GSH+GSSG_in _TEG: Total Extracellular Glutathione or GSH+GSSG_ex_

## Authors' contributions

VMU carried out the molecular cloning and glutathione assays, drafted manuscript. VMA carried out microbiological and fermentation experiments and helped to write the manuscript. AYM performed transcript analysis. HAK conceived the study, designed the experiment and helped to write the manuscript. AAS reviewed and edited the paper. All authors read and approved the final manuscript.
